# A Pain Obtunder

**Published:** 1887-06

**Authors:** F. H. Brimmer

**Affiliations:** Minneapolis


### A PAIN OBTUNDER.

BY F. H. BRIMMER, D. D. S., MINNEAPOLIS.

That rapid revolutions of the dental engine bur will act as a pain obtunder is not a new idea. That the principle has not been made to produce beneficial results is, I think, owing to the fact of the extent only to which the rule has been carried. Revolving the bur or drill rapidly, and *going ahead at the same time,* will not produce the desired result. I will cite one case in practice which will illustrate the manner in which rapid revolutions do actually lessen pain:

A lady desired to have the left superior central extracted ; the tooth was turned partly around in the socket, and so protruded as to present an unsightly appearance, though it was perfectly sound. As the tooth at the gum line was nearly round, it was easy to fit a Bonwill crown, and I advised her to have it cut off for that purpose. To cut off a tooth with a live pulp seemed indeed heroic practice, but after explaining how it could be done with little pain she submitted to the operation, and I proceeded as follows:

A large spear-shaped drill was held in light contact with the centre of the tooth at the gum line, and revolved rapidly for fifteen or twenty seconds, the contact being only sufficient to transmit vibrations. The drill was then pressed against the tooth with a little more force, and the sensibility being found diminished, it was allowed to penetrate perhaps one-third the distance to the pulp, when it was again used as at first. At the third trial the drill entered the pulp. With a probe, carbolic acid crystals were gradually forced to the end of the root, and in twenty-three minutes the tooth was cut off and properly shaped for the crown, which was set on the following day. The whole was accomplished almost without pain.

This need not be thought an exaggeration, for I think it can be satisfactorily explained. There is a limit to nerve conductibility. If a nerve be continuously irritated, after a time it loses its power of transmission until time has been given for it to recover its normal condition. The continual jarring of the drill exhausts the irritability of the nerve, and it no longer conveys sensation until it has had time to recover its tone. I hope that those who may read this article will experiment carefully, even though they may have no faith in this theory of a vibratory obtundent. I have been using in every-day practice rapid revolutions in the manner described for excavating sensitive teeth, such as usually are supposed to demand preparatory treatment to make the excavation bearable, and the result has been very satisfactory in every instance. I feel assured that there is something besides enthusiasm and imagination in this method of treating sensitive teeth for excavating, and for other operations which usually involve pain, and I am confident that the operator who employs it will find a real obtunder.

